# Psychoactive Substance Use and Its Relationship to Stress, Emotional State, Depressive Symptomatology, and Perceived Threat During the COVID-19 Pandemic in Mexico

**DOI:** 10.3389/fpubh.2021.709410

**Published:** 2021-08-23

**Authors:** Nora Angélica Martínez-Vélez, Marcela Tiburcio, Guillermina Natera Rey, Jorge Ameth Villatoro Velázquez, Miriam Arroyo-Belmonte, Graciela Yazmín Sánchez-Hernández, Morise Fernández-Torres

**Affiliations:** ^1^Department of Social Sciences in Health, Direction of Epidemiological and Psychosocial Research, Ramón de la Fuente Muñiz National Institute of Psychiatry, Mexico City, Mexico; ^2^Direction of Epidemiological and Psychosocial Research, Ramón de la Fuente Muñiz National Institute of Psychiatry, Mexico City, Mexico

**Keywords:** substance use, mood, mental health, COVID-19, Mexico

## Abstract

People can increase their use of psychoactive substances in response to stressful situations as a maladaptive mechanism for reducing negative affective states. It is therefore necessary to examine changes in the use of such substances and their relationship to mental health in light of the COVID-19 pandemic.

**Objective:** Evaluate the relationship between psychoactive substances and stress, emotional state, and symptomatology during the COVID-19 lockdown in Mexico.

**Method:** A national survey was conducted, using the free Google Forms platform, of residents of Mexico aged 18 and older. The survey was disseminated through social media.

**Results:** The sample comprised 4,122 individuals, mostly women (71.8%), with an age range of 18–81 years (*M* = 37.08, *SD* = 12.689), of which 46.8% were single, and 42.9% married. In general, there was a reduction in substance use during the first 2 months of the quarantine; the most commonly used substances were alcohol, tobacco, and tranquilizers. Respondents who described having greater use than before the pandemic presented greater stress, depressive symptomatology, and perceived threat than those who did not use substances.

**Conclusions:** Respondents who did not use substances reported lower levels of stress, depressive symptomatology, impact of the coronavirus pandemic, and perception of its threat. Women reported greater stress, depressive symptomatology, and emotional intensity than men.

## Introduction

Epidemiological studies of alcohol and other substance use show that the phenomenon varies over time. It is sometimes associated with stressful events such as economic crises (1–3), natural disasters (4), armed conflicts (5), and terrorist attacks (6). These and other studies show that such events play a key role in alcohol and other substance use, as well as mental health problems and somatic disorders (7–10).

The international community today faces a health crisis with the SARS CoV-2 pandemic, which is predicted to have a significant negative impact on the world economy (11) and the mental health of the population (12). In Mexico, the first case of COVID-19 was confirmed on February 28, 2020, and a National Healthy Distance Program was launched on March 23, recommending that the general population stay at home, and suspending in-person classes at all levels of education and non-essential activities in the public, social, and private sectors (13). The unique situation created by the COVID-19 pandemic has affected every country in the world and given rise to stressful phenomena such as depression, fear of the unknown nature of the disease and of being infected, vulnerability, requiring changes in daily life, working from home, anxiety about income, and the fear of losing one's job (14, 15). It has fostered negative emotional states with undesirable results for health and well-being, including changes in the use of alcohol, tobacco, and other drugs (16–18).

Although research addressing substance use in the context of the COVID-19 pandemic has emerged (16–19), earlier studies on other large-scale stressors suggest that substance use increases during exposure to disasters (20, 21). Some models postulate that an increase in negative affect in response to disasters increases the motivation to use substances as a coping mechanism to reduce tension, anxiety, and distress (10, 22, 23). Given the observed increase in anxiety, depression, and stress in response to COVID-19 (24–27), people may be using substances to cope with the negative affect accompanying this pandemic.

The data clearly call for an examination of the impact of highly stressful situations on substance use, and highlight the need to monitor variations in behavior during crises and offer interventions that will contribute to reducing their effects. A systematic review found several issues related to substance use that require special attention during the pandemic. These include an increase in mental health problems, a decrease in social interaction, and situations related to older adults, those aged 21–40 and persons in drug addiction treatment (28).

During stressful events, men and women cope with situations in different ways. Women tend to repress their emotions but seek help, while men attempt to resolve situations without help (29). At the same time, housework and the care of children and the elderly mainly falls to women, who experience a greater impact due to COVID-19 lockdown and stressful events related to the family, illness, and financial uncertainty (30). Other surveys applied during social isolation in the pandemic have found differences by gender, with women reporting a greater psychological impact and displaying higher levels of depression symptoms, anxiety, post-traumatic stress, and perceived loneliness than men in addition to an increase in the use of psychotropic drugs (31).

Differences by sex in the prevalence of substance use and abuse have significantly declined in the past three decades, which can be attributed to social and cultural factors that move women away from more traditional gender roles (such as employment opportunities and access to birth control) rather than biological sex differences (32, 33).

There are demographic, social, and cultural factors that disproportionately affect women and interact with the etiology and maintenance of use and substance use disorders, examples of which are care of children and the elderly and exposure to violence (32, 34).

Given that most research on substance use in the context of disasters has focused on predicting its increase (21, 35), there is also a need for studies that examine the differences in socioemotional factors in a context of fear and uncertainty regarding the pandemic, among those who used substances before it began, those who began use with the outbreak, those who did not change their patterns of use, and even some who reduced their use during the initial lockdown. We therefore sought to evaluate the relationship between stress, emotional state, depressive symptomatology, perception of threat from the coronavirus, and substance use during the first 3 months of lockdown in Mexico.

## Materials and Methods

The research protocol and data collection for this study were approved by the Ethics Committee of the Ramón de la Fuente Muñiz National Institute of Psychiatry (Approval No. CEI/C/011/2020), and participants gave their consent prior to taking the survey.

### Study Design

This was an exploratory, descriptive study using an online survey to explore substance use and the presence of mental health problems from March 23, 2020, the beginning of lockdown in Mexico.

### Participants

A total of 4,122 individuals were surveyed. All of them were aged 18 or over, residents of Mexico, and gave consent for their voluntary participation.

### Instruments

Although the questionnaire comprised 13 sections, this article only presents data on the following:

#### Sociodemographic Data

Ten questions on sex, age, education, marital status, occupation, state of origin, income, and family characteristics including total family members, number of children under 12, and number of older adults.

#### Perceived Threat and Experiences With Coronavirus

Short version of three scales developed by Conway et al. (36) that explore the perceived threat of coronavirus (three items, α = 0.89), the impact of coronavirus (six items, α = 0.84), and experiences with coronavirus (six items, α = 0.71). The scales, translated into Spanish for this study, have seven Likert responses ranging from 1 (“not true of me at all”) to 7 (“very true of me”).

#### Adversity and Stress Index

Eleven questions formulated for this study to measure the level of stress caused by the pandemic in different aspects of life during the previous month. The questions were divided into two groups: (a) relational stress, due to the effects on social interactions at school or work, or on leisure management (six items); and (b) contextual stress, associated with changes in a person's social and economic status (five items). There were five response options on a Likert scale, ranging from 0 (“not at all or only slightly stressful”) to 4 (“very stressful”). The reliability coefficient for this sample was 0.86.

#### Patient Health Questionnaire 2

The first two questions from the PHQ-9, which identify depressive symptomatology in the previous 2 weeks. There were four response options, ranging from 0 (“never”) to 3 (“almost every day”), and the maximum possible score was 6 (37). In Mexico, the discriminatory power of this questionnaire has been evaluated with indigenous women, and the best cutoff point found was 3, with a sensitivity of 80% and a specificity of 86.8% (38). The reliability coefficient for this sample was 0.78.

#### Substance Use

Based on the substance classification in ASSIST (39), this section explored the frequency of alcohol, tobacco, and other psychoactive substance use before and during lockdown, “How often did you use these substances BEFORE lockdown? SINCE LOCKDOWN STARTED, How often have you used these substances?” (The response options were never, once a month or less, 2–4 times a month, once a week, and daily or almost daily, for each of the substances.) with questions about experimentation with new substances during lockdown, perceived increase or decrease in substance use during this period, and possible reasons for these changes.

#### Emotional State

This section presented a list of 12 emotions, six positive and six negative, that may be experienced during quarantine, with five Likert responses ranging from 1 (“not at all”) to 5 (“a lot”).

### Procedure

The national online survey using Google Forms, conducted in May and June of 2020, was aimed at people aged 18 and over resident in Mexico. The link to the questionnaire was disseminated on the official social media accounts (Facebook and Twitter) of the Ramón de la Fuente Muñiz National Institute of Psychiatry, and by the research team using WhatsApp.

### Data Analysis

Data were analyzed with the statistical software IBM SPSS version 26. For the description of the sample by sex with the different sociodemographic indicators, the percentages were obtained and χ^2^ was used. Four groups were defined using the reports on substance use before and during lockdown: NU, non-users; NC, users who did not change their use during lockdown; DU, users who decreased their use during lockdown; and IU, users who increased their use during lockdown. These four groups, together with sex, were the comparison variables for each of the variables of interest (stress, emotional state, depressive symptomatology, and perceived threat). To control the variations between the groups and the continuous variables of interest, a multivariate analysis of variance was used. Although this statistical test assumes multivariate normality, several authors indicate that its results are valid even though this assumption is not fully met (40, 41). Additionally, in this analysis, the Bonferroni test was used to analyze the *post-hoc* comparisons between the four groups. Interactions were not included in the tables because only one of them was significant, which is indicated where applicable.

## Results

### Participant Characteristics

The sample consisted of 4,122 respondents, mostly women (71.8%), ranging in age from 18 to 81 years (*M* = 37.08*, SD* = 12.689), of which 46.8% were single, and 42.9% married. A large proportion had completed college (52.6%) or graduate (24.6%) education; 54.5% were employed, 14.6% self-employed, and 16.9% students. As shown in Table 1, there were statistically significant differences by sex for all the sociodemographic variables.

**Table 1:** Sociodemographic characteristics.

	**Men**		**Women**		**Total**		
	**(** ***n*** **=** **1,160)**		**(** ***n*** **=** **2,962)**		***N =*** **4,122**		
	***f***	**%**	***f***	**%**	***f***	**%**	**Chi square/** ***df***
**Age**
18–20 years	92	7.9	231	7.8	323	7.8	13.908^*^/4
21–30 years	326	28.1	832	28.1	1,158	28.1	
31–40 years	287	24.7	858	29.0	1,145	27.8	
41–50 years	230	19.8	588	19.9	818	19.8	
51 years or more	225	19.4	453	15.3	678	16.4	
**Marital Status**
Single	551	47.5	1,379	46.6	1,930	46.8	18.389^*^/3
Married/Partnered	525	45.3	1,245	42.0	1,770	42.9	
Divorced/Separated	78	6.7	292	9.9	370	9.0	
Widowed	6	0.5	46	1.6	52	1.3	
**Education**
Elementary/Jr. High	40	3.4	84	2.8	124	3.0	14.473^*^/3
High school	264	22.8	553	18.7	817	19.8	
Bachelor's degree	560	48.3	1,609	54.3	2,169	52.6	
Graduate degree	296	25.5	716	24.2	1,012	24.6	
**Occupation**
Homemaker	9	0.8	195	6.6	204	4.9	65.918^*^/5
Unemployed b/l	52	4.5	118	4.0	170	4.1	
Unemployed s/l	58	5.0	144	4.9	202	4.9	
Employed	640	55.2	1,607	54.3	2,247	54.5	
Student	200	17.2	498	16.8	698	16.9	
Self-employed	201	17.3	400	13.5	601	14.6	

*b/l, before lockdown; s/l, since lockdown*.

* *p ≤ 0.01*.

**Figure 1:**
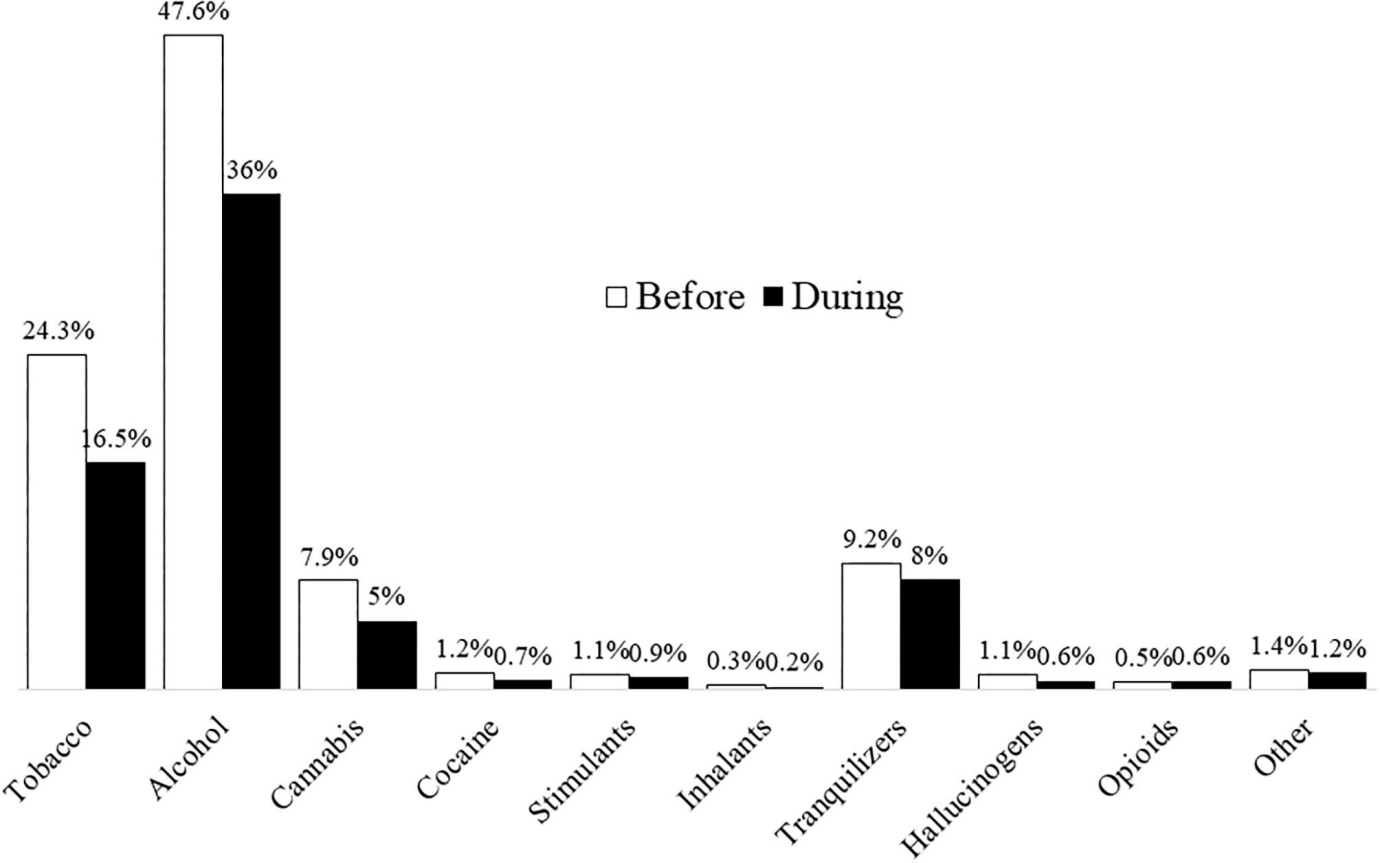
Substance use before and during the quarantine.

The prevalence of substance use before and during lockdown is shown in Figure 1. The highest percentages are seen for alcohol (47.6% before and 36% during the pandemic), tobacco (24.3% before and 16.5% during the pandemic), and non-prescription tranquilizers (9.2% before and 8% during the pandemic). The prevalence of other substance use was <8% and was not included in the rest of the analysis.

Table 2 shows the distribution of tobacco, alcohol, and tranquilizer use in the sample before and during the quarantine. The majority of participants were non-users. Among tobacco users, 11% reported no change, 11.3% a decrease, and 3.4% an increase in use. Among people who use alcohol, 18.1% reported no change, 19.7% a decrease, and 12.5% an increase in use. Among tranquilizer users, 3.6% reported no change, 3.7% a decrease, and 4.7% an increase in use.

**Table 2:** Distribution of tobacco, alcohol, and tranquilizer users^*^.

	**NU**		**NC**		**DU**		**IU**	
	***f***	***%***	***f***	***%***	***f***	***%***	***f***	***%***
Tobacco	3,064	74.2	455	11.0	467	11.3	141	3.4
Alcohol	2,050	49.7	748	18.1	814	19.7	515	12.5
Tranquilizers	3,635	88.1	149	3.6	151	3.7	192	4.7

*NU, non-user; NC, no change; DU, decreased use; IU, increased use*.

* *Percentages of total sample*.

### Relationship of Tobacco Use With Stress, Emotional State, Depressive Symptomatology, and Perceived Threat of Coronavirus

Respondents who did not use tobacco also showed significantly lower scores for relational and contextual stress than the other groups. On the relational stress subscales, there were significant differences between those who either increased or decreased their use and those who did not change; on the contextual stress subscales there were differences between those who did not change and those who increased their use. The comparison by sex showed that women experienced significantly greater stress than men (Table 3).

**Table 3:** Relationship of tobacco use and gender with stress, depressive symptomatology, emotional state, and perceived threat of coronavirus.

**Tobacco use**	**M**	**F**	***F (df = 1)***	**NU**	**NC**	**DU**	**IU**	***F (df = 3)***
Relational stress scale	6.49	7.82	32.498*	7.07^NC,DU,IU^	7.81^DU,IU^	8.91	9.73	22.458^*^
Contextual stress scale	7.22	8.67	28.826^*^	7.96^NC,DU,IU^	8.69^IU^	9.25	10.19	16.626^*^
Positive emotions	17.52	16.92	9.654^**^	17.11	17.12	17.12	16.53	
Negative emotions	16.77	19.58	84.744^*^	18.29^NC,DU,IU^	19.88^IU^	20.15^IU^	21.80	29.564^*^
Depressive symptomatology	1.86	2.34	49.915^*^	2.07^NC,DU,IU^	2.51^IU^	2.55^IU^	3.02	22.888^*^
Impact of coronavirus	15.97	16.22	7.810^**^	15.60^NC,DU,IU^	17.61^IU^	17.32	19.49	16.891^*^
Experiences with coronavirus	14.97	14.75	1.013	14.56^DU,IU^	14.81	15.98	16.31	4.627^**^
Perceived threat of coronavirus	8.63	9.97	16.450^*^	8.43^DU,IU^	9.45	10.40	10.83	7.353^*^

*The analysis used was a multivariate analysis of variance, using the four tobacco groups and gender as factors. The variables in the left column were the criterion variables. M, male; F, female; NU, non-user; NC, no change; DU, decreased use; IU, increased use. Group superscripts represent Bonferroni post-hoc significant differences between the group and the others. Only in the impact of the coronavirus scale was it found that women who increased their use had a higher mean*.

* *p ≤ 0.05*;

** *p ≤ 0.01*.

Although no significant differences were found between these groups with respect to positive emotions, the comparison by sex showed that men experienced these emotions more than women. Respondents who did not use tobacco experienced the fewest negative emotions. Those who increased their tobacco use showed more negative emotions than other groups, while women reported more negative emotions than men. The highest scores for depressive symptomatology were observed in women and in those who increased their tobacco use. The latter also perceived a greater impact of coronavirus than those who did not change their use; those who did not use tobacco perceived lesser impact than the rest. Women reported a greater impact than men. Those who increased and decreased their tobacco use described significantly more experiences and perceived threats of coronavirus than those who did not use tobacco. The comparison by sex only revealed differences with respect to the perceived threat. As for the interactions between sex and groups, none of them was statistically significant (Table 3).

### Relationship of Alcohol Use With Stress, Emotional State, Depressive Symptomatology, and Perceived Threat of Coronavirus

The group that increased its alcohol use reported significantly greater levels of relational and contextual stress than the other groups. Non-users of alcohol showed the lowest levels of stress, while women displayed more stress than men (Table 4). Those who reported no change in use showed a greater number of positive emotions than those who increased their use or did not use alcohol. Men reported significantly more positive emotions than women. Those who increased their alcohol use described more negative emotions than the other groups, while non-users reported the fewest of these emotions. Women experienced more negative emotions than men. Those who increased or decreased their alcohol use showed greater depressive symptomatology than those who did not change their use and those who did not use alcohol, while women showed more of these symptoms than men (Table 4). Those who increased their alcohol use showed significantly greater impact and experiences with coronavirus on both subscales than those who did not change their use or those who did not use alcohol. The perceived threat score was greater in those who increased their use than in the other three groups, and it was also greater in women. As for the interactions between sex and groups, only the women who increased their consumption, had a higher mean in the impact of coronavirus scale than the other combinations.

**Table 4:** Relationship between alcohol use and gender and stress, depressive symptomatology, emotional state, and perceived threat of coronavirus.

**Alcohol use**	**M**	**F**	***F (df = 1)***	**NU**	**NC**	**DU**	**IU**	***F (df = 3)***
Relational stress scale	6.49	7.82	59.185^*^	6.55^NC,DU,IU^	7.29^DU,IU^	8.60^IU^	9.42	41.732^*^
Contextual stress scale	7.22	8.67	63.277^*^	7.58^NC,DU,IU^	8.26^DU,IU^	8.88^IU^	9.99	29.626^*^
Positive emotions	17.52	16.92	7.445^**^	16.90^NC^	17.72^IU^	17.20	16.73	3.566^**^
Negative emotions	16.77	19.58	157.864^*^	17.76^NC,DU,IU^	18.77^DU,IU^	19.95^IU^	21.11	46.756^*^
Depressive symptomatology	1.86	2.34	64.736^*^	1.98^DU,IU^	2.11^DU,IU^	2.53	2.70	30.319^*^
Impact of coronavirus	15.97	16.22	3.636	15.24^DU,IU^	16.05^IU^	17.16	18.29	14.109^*^
Experiences with coronavirus	14.97	14.75	0.658	13.89^NC,DU,IU^	15.01^IU^	15.80	16.61	16.070^*^
Perceived threat of coronavirus	8.63	9.97	47.266^*^	9.03^DU,IU^	9.52^IU^	9.95^IU^	11.37	21.508^*^

*The analysis used was a multivariate analysis of variance, using the four alcohol groups and gender as factors. The variables in the left column were the criterion variables. M, male; F, female; NU, non-user; NC, no Change; DU, decreased use; IU, increased use. Group superscripts represent Bonferroni post-hoc significant differences between the group and the others. Interactions between sex and groups were not statistically significant*.

* *p ≤ 0.05*;

** *p ≤ 0.01*.

### Relationship of Tranquilizer Use With Stress, Emotional State, Depressive Symptomatology, and Perceived Threat of Coronavirus

Respondents who did not use tranquilizers showed significantly lower scores on the global stress scale as well as on the subscales. Those who increased their use had higher scores, as did women (Table 5). Non-users and men reported more positive emotions than the other groups. Women and those who increased their use had more negative emotions and depressive symptomatology than the others. Those who increased their use had significantly higher scores for the impact and perceived threat of coronavirus than the other groups. Non-users had significantly lower scores than the other groups. Women described a significantly greater perceived threat than men (Table 5). None of the interactions between sex and groups was statistically significant.

**Table 5:** Relationship between tranquilizer use and gender and stress, depressive symptomatology, emotional state, and perceived threat of coronavirus.

**Tranquilizer use**	**M**	**F**	***F (df = 1)***	**NU**	**NC**	**DU**	**IU**	***F (df = 3)***
Relational stress scale	6.49	7.82	5.930^*^	7.07^NC,DU,IU^	9.71	9.91	11.01	36.138^*^
Contextual stress scale	7.22	8.67	17.004^**^	7.91^NC,DU,IU^	11.19	10.33	11.27	28.700^*^
Positive emotions	15.52	16.92	4.017^*^	17.34^NC,DU,IU^	15.65	15.33	14.73	14.471^*^
Negative emotions	16.77	19.58	20.731^**^	18.32^NC,DU,IU^	21.41^IU^	21.40^IU^	23.62	44.113^*^
Depressive symptomatology	1.86	2.34	9.140^*^	2.07^NC,DU,IU^	3.01^IU^	3.07	3.54	48.240^*^
Impact of coronavirus	15.97	16.22	1.537	15.73^NC,DU,IU^	18.26^IU^	17.88^IU^	21.01	17.041^*^
Experiences with coronavirus	14.97	14.75	0.003	14.49^NC,DU,IU^	16.75	16.38	18.21	13.814^*^
Perceived threat of coronavirus	8.63	9.97	8.36^*^	9.35^IU^	10.55^IU^	10.05^IU^	13.03	18.994^*^

*The analysis used was a multivariate analysis of variance, using the four tranquilizer groups and gender as factors. The variables in the left column were the criterion variables. M, male; F, female; NU, non-user; NC, no change; DU, decreased use; IU, increased use. Group superscripts represent Bonferroni post-hoc significant differences between the group and the others. Interactions between sex and groups were not statistically significant*.

* *p ≤ 0.05*;

** * p ≤ 0.01*.

## Discussion

The purpose of this study was to explore changes in substance use during the COVID-19 lockdown in Mexico and their relationship with stress, depressive symptomatology, emotional state, and perceived threat of coronavirus. The results showed that alcohol, tobacco, and tranquilizers were the substances most commonly used during lockdown, but that there was a reduction in their use. This finding is similar to that reported by Manthey et al. (42) for various European countries, except that in their international survey, marijuana was the third most commonly used substance, after alcohol and tobacco. They believe their results could be partially explained by the reduced availability of substances during the early months of lockdown, as well as a change in the settings where they are used. This hypothesis could also explain the results of our analysis. In Mexico, substance use, especially by young people, generally occurs outside the home. According to Gómez et al. (43), young people prefer to use alcohol in bars and clubs (33.6%), friends' homes (20.7%), other public places like restaurants and schools (16.7%), and only 11.5% prefer to do so at home. The sale of alcohol has also been limited by the imposition of dry laws in several states, and the pandemic has had significant effects on family income. A study conducted in Spain (44) found that 21.5% of those surveyed reported having used tranquilizers in the previous month, 12% began using them during the pandemic, and one in three took more than the recommended dose or changed to a drug with stronger effects. Total use was greatest in women, similar to our own findings.

Another possible explanation for the increase in tranquilizer use may be related to problems of insomnia, in addition to those of anxiety, stress, and depression, as reported in a study in China (45).

Several studies conducted during the pandemic have focused mainly on the use of alcohol and tobacco and less so on other substance use. Our study found that non-prescription tranquilizers were the third most commonly used substances during lockdown. In Italy, an analysis of hair samples from drug users (46) found that heroin, cocaine, MDMA, and cannabis use dropped significantly, but that use of alcohol and benzodiazepines increased, probably because of their availability. This explanation could also apply to our findings.

Our finding of differences in depressive symptomatology between those who did not use alcohol or did not change their use and those who changed their use in response to lockdown coincides with the findings of studies conducted in the U.K., the U.S., and Australia (47–49). A similar relationship was observed with the perceived threat, impact, and experiences of coronavirus.

We found lower scores on the stress scale among those who did not use substances, while those who reported an increase in their use of alcohol showed significantly higher stress scores than those who reduced their use. Contextual stress factors, like the general social and economic situation, had a major impact on all the groups analyzed, particularly among those who increased their use of alcohol, tobacco, and tranquilizers. Studies in other countries suggest that high levels of stress could be related to increased use of alcohol and other substances as a maladaptive coping strategy (50), but our findings do not point in that direction.

In general, our respondents described experiencing negative emotions with great intensity. This tendency is clearest among those who use alcohol, tobacco, and tranquilizers, although the comparison by sex shows that women experience more negative emotions than men. This was also a finding of Ramos-Lira et al. (51), who investigated emotional responses and coping strategies during lockdown. They suggest that this difference may be the result of men's tendency to talk less about their emotions, part of the social expectations about masculinity that demand strength in the face of adversity, while women feel more freedom to express their feelings and negative emotions. Our findings support this observation.

We found more depressive symptomatology among respondents who used tobacco, as did Stanton et al. (49). As has been documented in research prior to the pandemic (52), tobacco is commonly used to cope with anxiety and depression. Since it is legal, there is a greater tolerance toward its use in the family environment and in crisis situations such as lockdown.

With respect to the limitations of our study, it is important to acknowledge that the design was not probabilistic. The data are drawn from a self-selected sample, which points to a possible bias in the characteristics of respondents and also limits its generalizability. For this reason, our analysis should be taken with caution.

## Conclusions

Non-substance users reported lower levels of stress, depressive symptomatology, impact of the coronavirus pandemic, and perceived threat of coronavirus. At the same time, women reported greater stress, depressive symptoms, and negative emotions than men. As in the surveys conducted during the Covid-19 lockdown, women reported an increase in tranquilizer use.

It is essential to develop mental health programs for early detection, intervention, and follow-up using communication and information technologies. These should actively consider patient opinions and individual traits examined in this study: women, negative emotions, substance use, and perceived threat of coronavirus.

## Data Availability Statement

The raw data supporting the conclusions of this article will be made available by the authors, without undue reservation.

## Ethics Statement

All procedures followed were in accordance with the standards of the Research Ethics Committee of the National Institute of Psychiatry (Approval No. CEI/C/011/2020). The participants provided their written informed consent to participate in this study.

## Author Contributions

NM-V, MT, and GNR contributed to the conception and design of the study. NM-V organized the database. NM-V, JVV, and MA-B performed the statistical analysis. MT and NM-V wrote the first draft of the manuscript. MA-B, JVV, GNR, GS-H, and MF-T wrote sections of the manuscript. All authors contributed to manuscript revision, read, and approved the submitted version.

## Conflict of Interest

The authors declare that the research was conducted in the absence of any commercial or financial relationships that could be construed as a potential conflict of interest.

## Publisher's Note

All claims expressed in this article are solely those of the authors and do not necessarily represent those of their affiliated organizations, or those of the publisher, the editors and the reviewers. Any product that may be evaluated in this article, or claim that may be made by its manufacturer, is not guaranteed or endorsed by the publisher.
